# Hepatitis B Virus, *Helicobacter pylori* and High-Risk Events of Gastric Cancer Development: An Observational Study (SIGES)

**DOI:** 10.3390/jcm15062413

**Published:** 2026-03-21

**Authors:** Jin-Chen Zou, Mao-Yao Wen, Yuan Yang, Zhuo-Yu Li, Yan Huang, Xin-Zu Chen, SIGES Research Group

**Affiliations:** 1 Gastric Cancer Center, Department of General Surgery, West China Hospital, Sichuan University, Chengdu 610041, China; 2 Department of General Surgery, Second People’s Hospital of Yibin City (West China Yibin Hospital), Sichuan University, Yibin 644000, China; 3 Health Management Center, General Practice Medical Center, West China Hospital, Sichuan University, Chengdu 610041, China; 4 Department of Gastrointestinal and Hernia Surgery, Second People’s Hospital of Yibin City (West China Yibin Hospital), Sichuan University, Yibin 644000, China; 5 Ya’an Cancer Prevention and Control Center, Ya’an People’s Hospital (West China Ya’an Hospital), Sichuan University, Ya’an 625000, China; 6 Ya’an Key Laboratory for High Altitude Medicine, Ya’an People’s Hospital (West China Ya’an Hospital), Sichuan University, Ya’an 625000, China

**Keywords:** gastric cancer, atrophic gastritis, gastrin, pepsinogen, hepatitis B virus, *Helicobacter pylori*

## Abstract

**Background:** Hepatitis B virus (HBV) is an infection proven to increase the risk of gastric cancer, especially among hepatitis B virus surface antigen (HBsAg) seropositive patients. However, the route through which HBV injures gastric mucosa and its mechanism of gastric carcinogenesis are still under investigation. **Aims:** The present study aimed to observe and evaluate associations between HBV infection with *Helicobacter pylori*, atrophic gastritis, and some other high-risk events for gastric cancer development. **Methods:** A retrospective cross-sectional study recruited participants undergoing a health check-up between 2018 and 2020 in the West China Hospital of Sichuan University. Participants were stratified into three statuses, including Group A (non-HBV infection), Group B (resolved HBV infection), and Group C (chronic HBsAg carriers or active HBV infection). Additionally, Groups A and B were categorized as HBsAg-seronegative, whereas Group C was defined as HBsAg-seropositive. High-risk events of gastric cancer included a history of gastric ulcer, *Helicobacter pylori* infection, serological atrophic gastritis (serum pepsinogens), hypergastrinemia (serum gastrin-17), and endoscopic findings of atrophic gastritis, gastric polyps, and gastric ulcer. Associations of HBV infection status or HBsAg seropositivity with *Helicobacter pylori* infection, atrophic gastritis and other high-risk events of gastric cancer were analyzed. **Results:** A total of 21,505 eligible observations were included, with Group C accounting for 6.1%. In Group C, the prevalence of gastric ulcer (*p* = 0.002) and very-high serum gastrin-17 level (*p* = 0.002) was significantly greater than in Group A. In multivariate analysis, both *Helicobacter pylori* infection (aOR = 2.79, 95% CI 2.44–3.21) and HBsAg seropositivity (aOR = 1.28, 95% CI 1.02–1.59) were significant risk factors for hypergastrinemia. No interaction was found between *Helicobacter pylori* co-infection risks and Group B (aOR = 1.10, 95% CI 0.84–1.43) or Group C (aOR = 1.40, 95% CI 0.66–2.95). *Helicobacter pylori* infection was identified as an independent risk factor for atrophic gastritis (aOR = 1.85, 95% CI 1.44–2.39). However, HBsAg seropositivity did not show a similar association with atrophic gastritis (aOR = 1.15, 95% CI 0.75–1.74). Moreover, HBV co-infection did not exert a synergistic effect on the risk of atrophic gastritis in individuals with *Helicobacter pylori* (aOR = 1.09, 95% CI 0.54–2.22). Additionally, multivariate analyses did not identify significant associations between HBV infection statuses and gastric polyps or ulcers. **Conclusions:** HBsAg seropositivity was not associated with increased risk of atrophic gastritis, gastric polyps or ulcers, or *Helicobacter pylori* infection, with the exception of hypergastrinemia. Additionally, HBV co-infection did not exert a synergistic effect on increasing the risk of atrophic gastritis in patients with *Helicobacter pylori*. Collectively, these findings suggest that the mechanism underlying the increased risk of gastric cancer in individuals with HBV may not be predominantly mediated via *Helicobacter pylori* infection and atrophic gastritis. Theories regarding HBV-induced genotoxicity or confounding effects warrant further investigation.

## 1. Introduction

Gastric cancer is one of the most highly prevalent malignancies in China, and the disease burden remains higher than the global level. Correa’s cascade theory is the classic inflammatory pathway for gastric carcinogenesis, namely the gradual development and evolution process from chronic gastritis, atrophic gastritis, intestinal metaplasia, and dysplasia to gastric cancer [1,2]. Common pathogens for gastric cancer are known to include *Helicobacter pylori* (*H. pylori*) and Epstein–Barr virus (EBV) [2,3]. Meanwhile, hepatitis B virus (HBV) infection is known as a global public health issue, involving around 296 million people around the world [4], and China is also an endemic area of HBV infections. It is known that HBV is characterized by its pantropic and oncogenic nature. Besides invading the liver and increasing the risk of liver cancer, it is also able to invade various extrahepatic tissues and increase the risk of extrahepatic cancers, especially digestive system cancers among adults [5].

Previous studies found that individuals with HBV, especially those seropositive for the hepatitis B virus surface antigen (HBsAg), had a significantly increased risk of gastric cancer [5,6]. The route through which HBV injures the gastric mucosa and its mechanism of gastric carcinogenesis are still under investigation. Therefore, it is primarily hypothesized that high-risk events of gastric cancer might be associated with HBV infection through classic inflammatory theory, particularly regarding *H. pylori* infection and atrophic gastritis, and we performed the present cross-sectional study to observe potential associations in the aspect of epidemiology. A key novelty of this study is its potential to identify or exclude certain intermediate events that could mediate the effect of HBV infection on the elevated risk of gastric carcinogenesis.

## 2. Methods

### 2.1. Study Design

This hospital-based retrospective cross-sectional study was conducted within serial studies of the Sichuan Gastric Cancer Early Detection and Screening (SIGES) research group. It was carried out in the West China Hospital, a central high-volume teaching hospital of Sichuan University. The period of the study was between 2018 and 2020. Health check-up observations were retrospectively collected at the Health Management Center, West China Hospital of Sichuan University. The study was briefly reported elsewhere with an identical design [7], but analyses were updated with a more restrained interpretation.

### 2.2. Ethics

This study retrospectively collected the records of participants’ general information and their test or examination results. SIGES serial studies obtained approval by the Biomedical Ethical Committee in West China Hospital of Sichuan University (identifiers: 2015-151-V2, 2 November 2015; 2018-215-V1, 6 August 2018), in accordance with the Declaration of Helsinki. In addition, informed consent was formally waived by the approval of the committee owing to the nature of retrospective observation, while anonymization of participants’ individual information was performed when analyzing and reporting data.

### 2.3. Eligibility and Groups

Cancer-free participants who underwent health check-ups were potentially eligible if they were 18–75 years old. Those with a history of malignancy or gastric surgery were excluded. A test of HBV serology was mandatory among all included participants. At least one high-risk event had to be assessed. Participants were classified into three groups according to HBV serologic statuses, including Group A who were not infected with HBV (HBsAg-, HBeAg-, anti-HBe-, and anti-HBc-, without or with anti-HBs+); Group B whose infection with HBV was treated (HBsAg-/HBeAg-, and at least anti-HBe+ or anti-HBc+, regardless of anti-HBs serostatus); and Group C which comprised chronic HBsAg carriers or patients with HBV (HBsAg+/HBeAg±, regardless of the serostatuses of anti-HBs, anti-HBe, and anti-HBc) [5].

### 2.4. Data Collection

The dataset was retrieved from electronic medical records with quality control, while cases with incomplete data were deleted. Demographic and baseline information included age, sex, education level, ethnicity, body mass index (BMI), family history of cancer, smoking status, and alcohol drinking status. Determinations included (1) the serology of gastrin-17 (G-17), pepsinogen-I (PG-I), pepsinogen-II (PG-II), HBV-specific antigens and antibodies; (2) a urea breath test for *H. pylori*, with a proton pump inhibitor, antibiotics, and bismuth withheld for at least 1 month; and (3) magnetically controlled capsule gastroscopy (MCCG). Eradication information was not available in the retrospective collection. At least one assessable outcome of the high-risk events was necessary, including comorbidities (liver cirrhosis, chronic gastritis, and self-reported gastric ulcer), *H. pylori* infection, serological atrophic gastritis (by serum pepsinogens), hypergastrinemia (by serum G-17), and MCCG findings including atrophic gastritis, gastric ulcers, and gastric polyps visually diagnosed by endoscopists [8,9,10]. Mild–moderate and severe atrophic gastritis were identified by serostatuses, respectively: (1) PG-I/II ratio < 3.0, and 20 ng/mL ≤ PG-I < 70 ng/mL, and (2) PG-I/II ratio < 3.0, and PG-I < 20 ng/mL [9]. A high level and very high level of serum G-17 were identified at >15 pmol/L and > 30 pmol/L, respectively [10].

### 2.5. Statistics

The prevalence rate of HBV infection (%) and the prevalence rates of high-risk events (per 1000 persons) were estimated. In the baselines, the Chi-square test was used for categorical parameters, while the Wilcoxon test was used for ranked parameters or continuous parameters where applicable. Associations of HBV infection with the prevalence of high-risk events for gastric cancer development and some other relevant risk factors were estimated in both univariate and multivariate analyses. Logistic regression was used for univariate and multivariate analyses to estimate the odds ratio (OR) and its 95% confidence interval (CI). The covariates of the regression model included sex, age, ethnicity, education level, BMI, smoking, drinking, and family history of malignancies. A complete case analysis was performed for missing data. All statistical tests were two-sided, and a *p* value < 0.05 was defined as statistically significant. The software STATA/MP 14.0 for Windows (32-bit) was used for statistical analysis.

### 2.6. Reporting

This retrospective cross-sectional study was qualified and reported according to the “Strengthening the Reporting of Observational Studies in Epidemiology (STROBE)” statement [11].

## 3. Results

### 3.1. Baselines and Risk Factors

**Figure 1:**
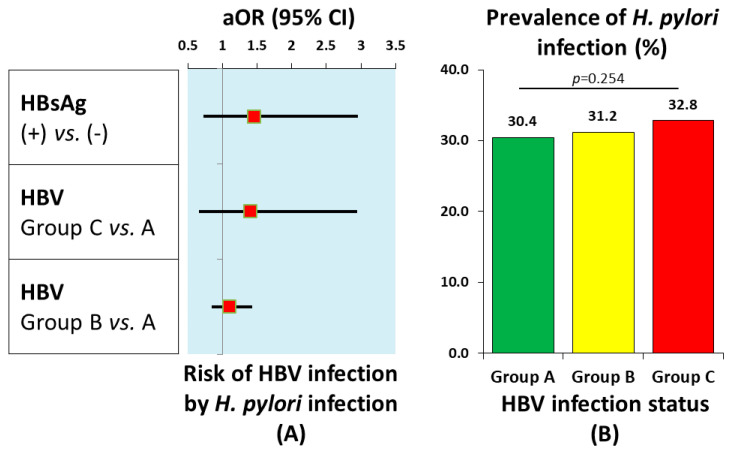
HBV and *H. pylori* co-infection. (**A**) Risk of HBV infection among individuals with *H. pylori* infection; (**B**) prevalence of *H. pylori* by HBV infection status.

A total of 21,505 participants were eligible and analyzed for this study, from which Groups B and C comprised 54.3% and 6.1%, respectively (Table 1). When compared to the baselines of Group A, the demographic and epidemiologic features of Groups B and C were significantly different, other than family history of cancer in Group B (*p* = 0.918) and ethnicity and alcohol drinking status in Group C (*p* = 0.061 and *p* = 0.316, respectively) (Table 1). Older age was an independent risk factor for HBV infection in the present study (Table 2). *H. pylori* infection was not associated with the risks of active or treated HBV infection (Table 2, Figure 1A).

### 3.2. Prevalence of High-Risk Events According to HBV Infection Status

In Group B, the prevalence rates of comorbid chronic gastritis (33.9 per 1000 persons vs. 25.0 per 1000 persons, *p* < 0.001), serologic atrophic gastritis (18.7 per 1000 persons vs. 13.9 per 1000 persons, *p* = 0.008), and very-high-level serum G-17 (28.6 per 1000 persons vs. 20.9 per 1000 persons, *p* < 0.001) were significantly greater than prevalence rates in Group A (Table 3). In Group C, prevalence rates of gastric ulcer (74.1 per 1000 persons vs. 7.9 per 1000 persons, *p* = 0.002) and very-high-level serum G-17 (28.9 per 1000 persons vs. 20.9 per 1000 persons, *p* = 0.002) were significantly greater than prevalence rates in Group A (Table 3). The prevalence of *H. pylori* infection was 32.5% in HBsAg-seropositive individuals (Group C), and was not significantly different among different HBV infection statuses (*p* = 0.254) (Table 3, Figure 1B).

### 3.3. Serologic Atrophic Gastritis, Hypergastrinemia, and H. pylori or HBV Infection Status

**Figure 2:**
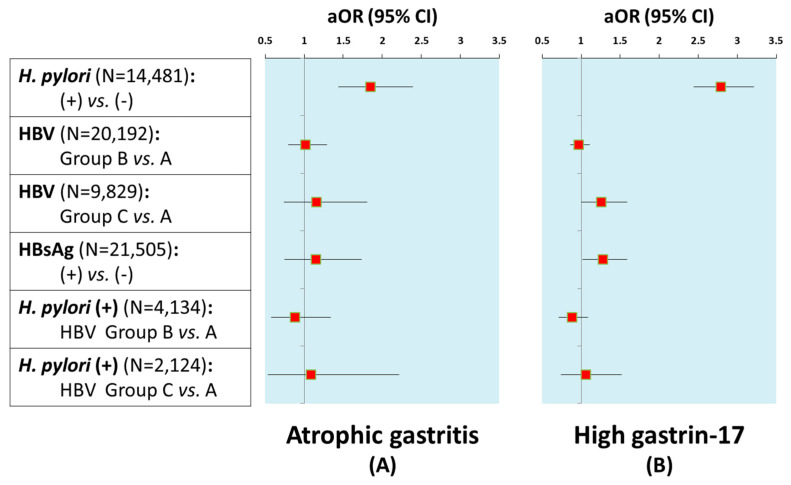
The risks of (**A**) atrophic gastritis and (**B**) high-level serum gastrin-17 associated with *H. pylori* and HBV infection statuses.

Active co-infection of *H. pylori* and HBV (HBsAg-seropositive status) merely accounted for 2.5%, while *H. pylori*-negative and HBsAg-seronegative status was found for the majority of participants (64.0%). Both univariate and multivariate analyses indicated that *H. pylori* infection was an independent risk factor of serologic atrophic gastritis (aOR = 1.85, 95% CI 1.44–2.39) (Table 4, Figure 2A). However, HBsAg seropositivity was not associated with serologic atrophic gastritis (aOR = 1.15, 95% CI 0.75–1.74) (Table 4, Figure 2A). Co-infection of HBV did not increase the risk of serological atrophic gastritis in individuals with *H. pylori* (aOR = 1.09, 95% CI 0.54–2.22) (Table 4, Figure 2A). Both *H. pylori* infection (aOR = 2.79, 95% CI 2.44–3.21) and HBsAg seropositivity (aOR = 1.28, 95% CI 1.02–1.59) were potential risk factors for a high level of serum G-17 (Table 5, Figure 2B). However, there was no additional synergistic effect by co-infection of HBV among individuals with *H. pylori* (aOR = 1.06, 95% CI 0.74–1.52) (Table 5, Figure 2B).

## 4. Discussion

This hospital-based cross-sectional study was performed based on the hypothesis that high-risk events might be associated with HBV infection. A total of 21,505 observations were analyzed, with 1432 cases undergoing MCCG. Older age was an independent risk factor for both HBsAg seropositivity and treated HBV infection. In Group C, the prevalence of gastric ulcer and very-high serum gastrin-17 levels was significantly higher than that in Group A. Multivariate analysis found that Group C and HBsAg seropositivity were significant risk factors associated with elevated serum G-17 levels, when compared to Group A and HBsAg seronegativity, respectively. HBV seropositivity was not associated with *H. pylori* infection, atrophic gastritis, gastric polyps or ulcers. *H. pylori* infection was identified as an independent risk factor of atrophic gastritis in the present study. Moreover, HBV co-infection did not exert a synergistic effect on the increasing risk of atrophic gastritis in individuals with *H. pylori* (aOR = 1.09, 95% CI 0.54–2.22). This indicates that HBV infection does not increase gastric cancer risk due to classic inflammatory carcinogenetic theory, i.e., Correa’s cascade.

Moreover, in recent decades, several epidemiologic studies have provided evidence that HBV infection is associated with the risk of gastric cancer. From our SIGES research group, both a previous hospital-based case–control study (aOR = 1.41, 95% CI 1.03–1.93) and meta-analysis (pOR = 1.39, 95% CI 1.11–1.75) found consistent results and a significant association between HBV infection and gastric cancer risk, especially among the HBsAg seropositive subset [5,6]. Additionally, Wu found that HBV infection was significantly associated with gastric cancer (OR = 2.21, 95% CI 1.21–3.47) [12]. Similarly, Wei found that HBsAg seropositivity was significantly associated with gastric cancer risk (aOR = 1.49, 95% CI 1.06–2.10) [13]. Song found similar associations of HBsAg seropositivity in the China Kadoorie Biobank prospective cohort study (HR = 1.41, 95% CI 1.11–1.80), and validated the associations of HBV infection in the Qidong cohort (HR = 2.02, 95% CI 1.24–3.29) and the Changzhou nested case–control study (OR = 1.76, 95% CI 1.04–2.98) [14]. Tian found that the risk of gastric cancer was elevated for those exposed to HBsAg seropositivity in both their case–control study (aOR = 1.46, 95% CI 1.30–1.65) and meta-analysis (pOR = 1.23, 95% CI 1.14–1.33) [15]. Yu similarly found a positive association between HBV infection and increased risk of gastric cancer development (pOR = 1.29, 95% CI 1.16–1.44) [16]. Wongtrakul found a significantly increased risk of incident gastric cancer among patients with chronic HBV infection in a meta-analysis (pRR = 1.49, 95% CI 1.20–1.85) [17]. In a Mendelian randomization study, genetic liability to chronic HBV infection was indicated to be causally associated with gastric cancer (OR = 1.12, 95% CI 1.05–1.19) [18]. In short, this evidence substantially indicates the epidemiologic association between infection with HBV and increased risk of gastric cancer development.

These findings may provide important implications for global public health policies on cancer prevention and screening strategies, while the need for multisystem cancer screening might be considered in subpopulations with HBV infection as a modifiable risk factor [19]. Moreover, current evidence strongly advocates further investigation and understanding of the potential mechanisms related to HBV infection, increasing the risk of gastric cancer. HBV-associated gastric cancer (HBVaGC), similar to HBV-induced liver cancer, is a complex process involving the interaction of multiple factors. The potential mechanisms for this process need to be studied.

First, HBV infection might induce inflammatory injury of the gastric mucosa and, consequently, initiate Correa’s cascade and other high-risk events. Wu found that HBV was significantly associated with gastric ulcers (OR = 10.51, 95% CI 5.66–19.52) [12]. HBV DNA and HBsAg levels were strongly correlated with the manifestation of gastritis and gastric ulcers, and were the highest in gastric cancer patients [12]. Kartsonaki found that HBV was associated with a higher risk of duodenal ulcer (HR = 1.46, 95% CI 1.04–2.05) in a case–cohort study from the China Kadoorie Biobank [20]. In a cross-sectional study, Baghbanian found that HBsAg was significantly associated with gastric precancerous histopathology (OR = 21.56, 95% CI 7.00–65.74) and as a risk factor for gastritis (OR = 12.46, 95% CI 3.01–51.61) [21]. However, similar results were not found in the present study. Prevalence of chronic gastritis, atrophic gastritis, and hypergastrinemia was increased in resolved HBV infection, while a higher prevalence of hypergastrinemia and gastric ulcer was found in active HBV infection. Nevertheless, multivariate analysis did not validate an association between HBsAg seropositivity and atrophic gastritis. Therefore, these findings partially refute the hypothesis that HBV infection might induce gastric cancer through the inflammatory mechanism, but limited evidence means these findings still warrant consideration with caution.

Second, HBV infection might provide a synergistic interaction for *H. pylori* infection and other confounders related to high-risk events and gastric carcinogenesis. Wu found that HBV infection potently increases the risk of gastric cancer development among patients with *H. pylori* (OR = 3.39, 95% CI 1.71–6.12) [12]. Baghbanian found that HBV infection significantly increased development of gastric premalignant conditions and cancer in patients with *H. pylori* [21]. However, our study found that neither active (aOR = 1.09, 95% CI 0.54–2.22) nor resolved (aOR = 0.88, 95% CI 0.58–1.34) infection of HBV increased the risk of atrophic gastritis among individuals with *H. pylori*. Chen found that smoking was associated with increased risk of gastric cancer, which was especially enhanced in the HBsAg-positive subset; however, alcohol drinking did not show a similar interaction with HBsAg seropositivity [22]. Our results found that the proportions of current or those with a history of smoking were significantly higher among active or resolved HBV infections, but smoking was not an independent risk factor for HBV infection. Whether HBV infection (as a confounder) increases the risk of high-risk events and gastric cancer due to synergistic interactions with definite risk factors needs further investigation.

Third, HBV infection might cause genotoxicity against host epithelial cells of the gastric mucosa, consistent with HBV-induced liver cancer [23]. The integration of HBV DNA into the host genome has commonly been considered an important and main mechanism for HBV-associated carcinogenesis of extrahepatic cancers [24]. Namely, integration of HBV may increase genomic instability and structural variations in gastric cancer, such as enriched CpG islands, and a raised proportion of deletions and insertions [24]. The HBx gene is essential for HBV replication and is expressed in the extrahepatic organs or tissues of patients with HBV [25]. HBx is closely associated with the development of gastric cancer, as it evades the surveillance of the immunologic system by integrating its sequence into the human genome [25]. Histology demonstrated that gastric HBx-positive epithelial cells had a higher nuclear–cytoplasmic ratio, compared to HBx-negative cells [26]. HBV infection was associated with gastric cancer risk in Mendelian randomization and bioinformatics analyses, through the evolutionary mechanism related to Toll-like receptor signaling and PI3K-Akt signaling [27]. Unique mutation profiles were found in HBV- gastric cancer specimens, including five frequently mutated protein-coding genes: FGF12, KMT2B, SOX1, KMT2D, and TUBB2B [28]. However, despite the latest information above, the causal relationship between HBV and gastric cancer and its biological mechanisms remains indeterminate and warrants further investigation.

Namely, the results of the present study are feasible, internally coherent, and consistent with current biological understanding. This study should be regarded as a clarifying negative study rather than a failed attempt to detect an association. It should be highlighted that this study meaningfully narrows the plausible mechanistic pathways linking HBV infection to gastric cancer development and discourages oversimplified inflammatory interpretations. Moreover, the reason why the epidemiological association between HBV infection and gastric cancer development persists despite the absence of intermediate gastric lesions in this study requires further clarification. As aforementioned, both the confounder theory and the genotoxicity theory should be considered and cannot be ruled out. More comprehensive studies on the associations between HBV infection and harmful or protective factors for gastric cancer are warranted, such as lifestyle, dietary habits, environmental exposure, and socioeconomic status. Big data and real-world studies can be applied to clarify whether HBV infection acts as a confounder in gastric carcinogenesis. Additionally, primary evidence for HBV genotoxicity in gastric mucosal epithelial cells is needed, which can be examined in non-cancerous and cancerous cells using in situ hybridization and single-cell sequencing. Inter-population heterogeneity among HBV genotypes and sub-genotypes should be investigated in subgroup analyses to identify highly carcinogenic genotypes and sub-genotypes [29]. Furthermore, the genetic susceptibility and heterogeneity of host epithelial cells also warrant future investigation.

There are several limitations that need to be considered with caution. First, this study was based on hospital-based retrospective cross-sectional participants, mainly composed of an urban population, rather than a general population. As such, there might be some sampling bias. Second, the proportion of participants undergoing MCCG was relatively low due to the nature of the health check-up series and was as low as 6.7% (1432/21,505). This low proportion can also introduce potential sampling bias because of the relatively low number of events. Therefore, multivariate regression was not performed in the MCCG subset. Third, the identification of atrophic gastritis was dependent on serology or MCCG, rather than biopsy and pathology. Some degree of misclassification might have influenced the present study. Fourth, in the present study, there was no case with severe atrophic gastritis diagnosed by serology. An additional observational study focused on severe atrophic gastritis alone might be a meaningful stratified study. Fifth, the major results appeared to show significant associations with a high level of serum G-17. However, a high level of serum G-17 cannot be a direct and strong interpretation of the carcinogenic mechanism for developing gastric cancer. Finally, information on genotypes of HBV and virulent strains of *H. pylori* was absent from the present study due to its retrospective nature, which may influence gastric pathology and may further dilute population-level associations if not accounted for. Namely, the confirmation of these associations should be done carefully, requiring more robust investigations.

## 5. Conclusions

HBsAg seropositivity was not associated with an increased risk of atrophic gastritis, gastric polyps or ulcers, as well as *Helicobacter pylori* infection, with the exception of hypergastrinemia. Moreover, HBV co-infection did not exert a synergistic effect on the increased risk of atrophic gastritis in individuals with *Helicobacter pylori*. Collectively, these findings suggest that the mechanism underlying the increased risk of gastric cancer in individuals with HBV may not be predominantly mediated via *Helicobacter pylori* infection and atrophic gastritis. Theories regarding HBV-induced genotoxicity or confounding effects warrant further investigation.

## Figures and Tables

**Table 1:** Demographic and epidemiologic features observed for HBV infection.

Variable	Group A		Group B		*p* Value * (B vs. A)	Group C		*p* Value *
	*n*	Proportion (%)	*n*	Prevalence (%)		*n*	Prevalence (%)	(C vs. A)
Sum	8516	39.6	11,676	54.3		1313	6.1	
Sex					<0.001			<0.001
Female	4074	35.4	6696	58.1		748	6.5	
Male	4442	44.5	4980	49.9		565	5.7	
Age (yrs)					<0.001			<0.001
≤30	1535	81.7	278	14.8		67	3.6	
≤40	2362	53.5	1762	39.9		290	6.6	
≤50	2407	33.2	4352	60.0		496	6.8	
≤60	1604	29.8	3445	64.1		329	6.1	
≤70	507	24.4	1459	70.2		112	5.4	
>70	101	20.2	380	76.0		19	3.8	
Ethnics					<0.001			0.061
Han	2592	36.5	4346	61.2		163	2.3	
Minority	137	49.8	135	49.1		3	1.1	
Education level					<0.001			<0.001
<Bachelor	1032	29.4	2393	68.2		84	2.4	
≥Bachelor	1347	49.5	1314	48.3		59	2.2	
BMI (kg/m^2^)					<0.001			0.007
Normal (≥18.5, <25)	5395	40.9	6992	53.1		795	6.0	
Underweight (<18.5)	606	61.0	336	33.8		51	5.1	
Overweight (≥25, <30)	2168	33.7	3860	60.0		410	6.4	
Obese (≥30)	318	39.4	440	54.3		51	6.3	
Smoking					<0.001			<0.001
Never	6076	41.1	7821	53.0		871	5.9	
Current or ever	2246	35.7	3625	57.7		413	6.6	
Alcohol drinking					<0.001			0.316
Never	4687	41.8	5790	51.6		739	6.6	
Current or ever	3660	37.1	5671	57.4		543	5.5	
Family history of cancer					0.918			0.006
None	7096	39.7	9743	54.4		1057	5.9	
Other malignancies	997	41.1	1277	52.6		153	6.3	
Gastric cancer	134	35.6	218	57.8		25	6.6	
Liver cancer	161	34.0	258	54.4		55	11.6	

Abbreviations: BMI, body mass index; HBV, hepatitis B virus. * *p* values for comparisons of Groups B or C to Group A, respectively, where the Chi-square test or Wilcoxon test is applicable.

**Table 2:** The association between risk factors and HBV infection status.

Factors	HBsAg+ vs. HBsAg-		Group C vs. Group A		Group B vs. Group A	
	(*n* = 6132)		(*n* = 2480)		(*n* = 5989)	
	aOR	95% CI	aOR	95% CI	aOR	95% CI
Sex						
Female	Ref.		Ref.		Ref.	
Male	1.03	(0.63–1.67)	0.82	(0.50–1.35)	0.70	(0.59–0.82)
Age (yrs)						
≤30	Ref.		Ref.		Ref.	
≤40	2.13	(0.73–6.21)	3.20	(1.10–9.32)	4.20	(3.13–5.65)
≤50	3.48	(1.24–9.78)	7.49	(2.67–20.99)	8.27	(6.19–11.05)
≤60	2.62	(0.92–7.52)	6.68	(2.33–19.14)	10.50	(7.81–14.10)
≤70	1.40	(0.40–4.93)	3.89	(1.10–13.82)	11.67	(8.29–16.42)
>70	2.64	(0.57–12.26)	8.08	(1.67–39.14)	13.93	(8.24–23.56)
Ethnics						
Han	Ref.		Ref.		Ref.	
Minority	0.63	(0.20–2.00)	0.45	(0.14–1.47)	0.56	(0.41–0.76)
Education level						
<Bachelor	Ref.		Ref.		Ref.	
≥Bachelor	1.00	(0.70–1.43)	0.70	(0.49–1.01)	0.53	(0.48–0.60)
BMI (kg/m^2^)						
Normal (≥18.5, <25)	Ref.		Ref.		Ref.	
Underweight (<18.5)	1.65	(0.74–3.68)	1.30	(0.57–2.94)	0.68	(0.49–0.94)
Overweight (≥25, <30)	1.04	(0.70–1.54)	1.19	(0.79–1.79)	1.16	(1.02–1.32)
Obese (≥30)	1.36	(0.61–3.03)	1.60	(0.79–3.70)	1.11	(0.83–1.48)
Smoking						
Never	Ref.		Ref.		Ref.	
Current or ever	1.45	(0.94–2.25)	1.38	(0.88–2.17)	0.95	(0.82–1.09)
Alcohol drinking						
Never	Ref.		Ref.		Ref.	
Current or ever	0.64	(0.42–0.99)	0.63	(0.40–0.99)	0.99	(0.85–1.14)
Family history of cancers						
None	Ref.		Ref.		Ref.	
Other malignancies	1.50	(0.96–2.34)	1.39	(0.88–2.21)	0.86	(0.73–1.02)
Gastric cancer	1.12	(0.35–3.60)	1.20	(0.36–3.99)	1.07	(0.71–1.61)
Liver cancer	1.63	(0.65–4.08)	1.44	(0.56–3.72)	0.76	(0.53–1.10)
*H. pylori* infection						
Negative	Ref.		Ref.		Ref.	
Positive	1.46	(0.72–2.96)	1.40	(0.66–2.95)	1.10	(0.84–1.43)

Abbreviations: BMI, body mass index; CI, confidence interval; HBV, hepatitis B virus; aOR, adjusted odds ratio; Ref., reference.

**Table 3:** Prevalence of high-risk events for gastric cancer according to HBV infection status.

Events	Group A		Group B		*p* Value *	Group C		*p* Value *
	*n*	Prevalence (per 1000 Persons)	*n*	Prevalence (per 1000 Persons)	(B vs. C)	*n*	Prevalence (per 1000 Persons)	(C vs. A)
Comorbidity (*n* = 21,050)								
Liver cirrhosis	1	0.1	5	0.4	0.207	11	8.6	<0.001
Chronic gastritis	208	25.0	388	33.9	<0.001	34	26.5	0.752
Ever gastric ulcer	77	9.3	111	9.7	0.749	10	7.8	0.606
*H. pylori* status (*n* = 14,481)								
Infected	1768	304.3	2366	311.9	0.343	356	328.4	0.114
Serologic atrophic gastritis (*n* = 21,505)								
Mild-moderate	118	13.9	218	18.7	0.008	24	18.3	0.211
Severe	0	0	0	0		0	0	
Serum Gastrin-17 (*n* = 21,505)					<0.001			0.002
High level	283	33.2	381	32.6		57	43.4	
Very high level	178	20.9	334	28.6		38	28.9	
MCCG findings (*n* = 1432)								
Atrophic gastritis	4	7.9	12	13.3	0.359	0	0	0.643
Polyp	25	49.5	53	58.9	0.461	1	37.0	0.770
Gastric ulcer	4	7.9	15	16.7	0.173	2	74.1	0.002

Abbreviations: HBV, hepatitis B virus; MCCG, magnetically controlled capsule gastroscopy. * *p* values for comparisons of Groups B or C to Group A, respectively, where the Chi-square test or Wilcoxon test is applicable.

**Table 4:** The association between serological atrophic gastritis and *H. pylori* or HBV infection status.

Factors	Univariate		Multivariate	
	OR	95% CI	aOR	95% CI
*H. pylori* infection (*n* = 14,481)				
Negative	Ref.		Ref.	
Positive	1.88	(1.46–2.42)	1.85	(1.44–2.39)
HBV infection (*n* = 21,505)				
Group A	Ref.		Ref.	
Group B	1.35	(1.08–1.70)	1.02	(0.80–1.29)
Group C	1.33	(0.85–2.06)	1.16	(0.74–1.81)
Serum HBsAg (*n* = 21,505)				
Negative	Ref.		Ref.	
Positive	1.10	(0.72–1.67)	1.15	(0.75–1.74)
Co-infection status (*n* = 4490)				
*H. pylori*+/HBV-	Ref.		Ref.	
*H. pylori*+/HBV Group B+	1.11	(0.75–1.66)	0.88	(0.58–1.34)
*H. pylori*+/HBV Group C+	1.22	(0.60–2.45)	1.09	(0.54–2.22)

Abbreviations: CI, confidence interval; HBV, hepatitis B virus; *H. pylori*, *Helicobacter pylori*; OR, odds ratio; Ref., reference.

**Table 5:** The association between a high level of serum gastrin-17 and *H. pylori* or HBV infection status.

Factors	Univariate		Multivariate	
	OR	95% CI	aOR	95% CI
*H. pylori* infection (*n* = 14,481)				
Negative	Ref.		Ref.	
Positive	2.81	(2.45–3.23)	2.79	(2.44–3.21)
HBV infection (*n* = 21,505)				
Group A	Ref.		Ref.	
Group B	1.14	(1.01–1.29)	0.97	(0.86–1.11)
Group C	1.36	(1.08–1.71)	1.26	(1.00–1.59)
Serum HBsAg (*n* = 21,505)				
Negative	Ref.		Ref.	
Positive	1.26	(1.02–1.57)	1.28	(1.02–1.59)
Co-infection status (*n* = 4490)				
*H. pylori*+/HBV-	Ref.		Ref.	
*H. pylori*+/HBV Group B+	0.96	(0.78–1.17)	0.88	(0.71–1.09)
*H. pylori*+/HBV Group C+	1.10	(0.77–1.58)	1.06	(0.74–1.52)

Abbreviations: CI, confidence interval; HBV, hepatitis B virus; *H. pylori*, *Helicobacter pylori*; OR, odds ratio; Ref., reference.

## Data Availability

In-house data will be available upon reasonable request by emailing the corresponding author chenxinzu@scu.edu.cn.
